# Family violence in a sample of treatment-seeking gamblers: the effect of having dependent children

**DOI:** 10.1186/s40405-017-0028-1

**Published:** 2017-10-13

**Authors:** Maria Bellringer, Janet Pearson, Katie Palmer du Preez, Denise Wilson, Jane Koziol-McLain, Nick Garrett, Max Abbott

**Affiliations:** 1 Gambling and Addictions Research Centre, Faculty of Health and Environmental Sciences, Auckland University of Technology, Private Bag 92006, Auckland, 1142 New Zealand; 2 Department of Biostatistics and Epidemiology, Faculty of Health and Environmental Sciences, Auckland University of Technology, Private Bag 92006, Auckland, 1142 New Zealand; 3 Interdisciplinary Trauma Research Centre, Faculty of Health and Environmental Sciences, Auckland University of Technology, Private Bag 92006, Auckland, 1142 New Zealand; 4 Taupua Waiora Centre for Māori Health Research, Faculty of Health and Environmental Sciences, Auckland University of Technology, Private Bag 92006, Auckland, 1142 New Zealand

**Keywords:** Problem gambling, Family violence, Dependent children

## Abstract

This study investigated the effect of problem gambler gender on the relationship between the gambler having dependent children (younger than 18 years) living at home and the gambler perpetrating or being a victim of family violence. The sample comprised 164 help-seeking gamblers (43% female; 37% with dependent child/ren) recruited from three national gambling treatment services in New Zealand. Family violence was measured using a modified version of the HITS scale covering physical, psychological, verbal, emotional and sexual violence. Forty-nine percent of participants reported being a victim of violence and 43% had perpetrated violence. Multivariable logistic regression modelling was conducted, adjusting in sequence for significant socio-demographic, psychosocial and gambling factors. The relationship between having dependent children and being a victim of family violence was gender-related. Female gamblers living with dependent children reported more family violence perpetration and victimisation than male gamblers living with dependent children. Female gamblers with dependent children living at home had greater odds of being a victim of family violence than male gamblers without dependent children living at home. This relationship remained when adjusted for contextual factors of being a victim (ethnicity, income support status, and feelings of inadequacy) in this sample. A similar gender effect of having dependent children living at home on violence perpetration disappeared when known psychosocial contextual factors of violence perpetration (aggression, difficulties in emotion regulation, drug issue in the family, and interpersonal support) were taken into account. These findings suggest the value of coordinated approaches between gambling treatment services and programmes supporting vulnerable families in order to identify vulnerable families and put support mechanisms in place.

## Background

Families with problem gamblers are families under pressure. International studies highlight the multifaceted and complex nature of the impacts of problem gambling on family dynamics. The most common problems reported by family members of problem gamblers have the potential to contribute to a home environment characterised by discord and deprivation: the loss of household or personal money; arguments, anger and violence; lies and deception; neglect of family; negatively affected relationships; poor communication; confusion of family roles and responsibilities; and the development of gambling problems or other addictions within the family (Kalischuk et al. 2006). A recent systematic literature review of 30 empirical studies examining the family impacts of gambling conducted between 1998 and 2013 identified: strain and conflict in family relationships; loss of trust; financial devastation; high levels of distress, anxiety and depression; physical health problems; and isolation from friends and wider family (Kourgiantakis et al. 2013). While most research has focused on the spouse/partner of a gambler, some research has specifically documented adverse effects on children such as experiences of parental physical and emotional unavailability, estrangement from wider family networks, loss of safety, material and financial deprivation, depressive symptoms and conduct problems (Kourgiantakis et al. 2013).

Family violence and problem gambling issues share a conceptualisation from broad public health and socio-political perspectives. They are both regarded as complex issues, affected by social and economic factors with inequalities in power and resources (e.g. between men and women, between socio-economic and cultural groups) playing a significant role. A recent multilevel investigation of correlates of intimate partner violence (IPV) in population-based datasets across 44 countries has shown that in addition to age, education and socio-economic status, gender inequality at the macro-level helps to predict population levels of IPV (Heise and Kotsadam 2015). Macro-level factors including the rights and societal positioning of indigenous groups are likely to be important determinants of IPV (United Nations 2014). The disproportionate gambling harm experienced by certain groups of people (conceived as ‘vulnerable’) is held to be related to their: socio-economic and political positioning within society (e.g. deprivation, lack of representation); access to gambling venues; processes of colonisation; cultural beliefs, values and practices; and migration and acculturation (Raylu and Oei 2004; Rintoul et al. 2013). Gender asymmetry in relation to norms and roles in society has also been linked to gambling practices and the way that responses to gambling (e.g. treatment and research) are enacted (Delfabbro 2000; Griffiths and Delfabbro 2001).

In New Zealand, despite limitations of available datasets (Family Violence Death Review Committee 2014; Gulliver and Fanslow 2013), research suggests that Māori have increased risk for family violence (e.g. Fanslow et al. 2010; Marie et al. 2008). For example, Marie, Fergusson and Boden (2008) found that (after controlling for socio-economic status, family functioning factors and individual factors) Māori males and females were at higher risk of being victims and perpetrators of IPV, as well as at higher risk of injuries related to IPV, than were non-Māori participants. Recent reviews have also raised the possibility of culturally specific factors that relate to family violence among Pacific and Asian people (Keen et al. 2015; Lievore et al. 2007). While the causes of family violence for Māori are acknowledged as a complex mix of historical and contemporary factors, the “magnitude and severity” of violence within whānau (extended family) is of epidemic proportions. It is argued that the violence has become normalised and tolerated despite being “the language of the powerless” (Kruger et al. 2004, p. 9).

In New Zealand, gambling harm is also particularly notable in relation to Māori, Pacific people and Asian communities (including recent migrant groups such as Chinese and Koreans) (Abbott et al. 2014). Māori have particularly high problem gambling prevalence rates relative to non-Māori (Abbott et al. 2014; Abbott and Volberg 1991, 1996, 2000; Ministry of Health 2006, 2008) and other populations studied internationally (Volberg and Abbott 1997). Māori (and Pacific) people have continued to be disproportionately affected since 1991 (Abbott et al. 2014). Similar to family violence, gambling and problem gambling are discussed in relation to broad impacts of colonisation (Watene et al. 2007). The accessibility of gambling products in low income communities, where many Māori and Pacific people reside, has been consistently noted (Clarke et al. 2006; Dyall 2007). Additionally, gambling activities are an accepted form of fund-raising to meet cultural responsibilities (Morrison and Wilson 2015; Perese 2009).

Population survey research has showed a considerable commonality in the socio-demographic factors found to produce vulnerability to gambling issues and family violence. These factors include: a low level of education; receiving government benefits; and consuming alcohol and drugs (Bohn et al. 2004; Fox and Benson 2006; Lown et al. 2006; McMillen and Marshall 2004; Wenzel et al. 2004). Notably, the marital status of problem gamblers has been found to be more likely to be ‘separated’ or ‘divorced’ (McMillen and Marshall 2004), indicating an obvious breakdown in family relationships.

The particular challenges of parenting children living at home are recognised as having the potential to place additional strain on vulnerable families when a gambler is present. In an early study, 10% of a sample of Gamblers Anonymous members reported that their problem gambler partner or spouse had abused their children (Lorenz and Shuttlesworth 1983). Lesieur and Rothschild (1989) found that children of Gamblers Anonymous members were more likely to have experienced parental violence and abuse (e.g. throw something; slap or spank; kick, bite or hit with a fist; and hit or try to hit with something) than a nationally normed sample. In their study, parents with gambling problems that were co-existent with other stress-related conditions such as alcohol dependence, substance abuse or over-eating behaviour, were more likely to be violent and abusive toward their children than problem gambler parents without these additional issues. A community survey in the Canadian province of Alberta revealed that diagnosed pathological gamblers reported higher rates of spousal abuse (hitting or throwing things more than once at spouse or partner) (23%) and child abuse (hitting child) (17%) than the general population (Bland et al. 1993). In a nationally representative sample of adults from the United States of America (USA), pathological gambling was associated with increased odds of perpetrating child abuse even after controlling for socio-demographic variables and mental disorders (Afifi et al. 2010).

Despite commonalities in theory and conceptualisation, there is a paucity of research establishing and exploring violence in families where there are gambling issues (Dowling et al. 2016). This is despite reports of high co-occurrence from practitioners across family violence, problem gambling and family/financial counselling services (for an Australian context see Dowling et al. 2014). No research specifically investigating this relationship has been conducted in New Zealand to date. The characteristics of families experiencing problem gambling have not been explored in detail, being largely limited to studies of family impacts and family coping (e.g. Dowling et al. 2009; Suomi et al. 2013).

Recent Australian research has explored the patterns and prevalence of family violence among treatment-seeking problem gamblers and family members (Dowling et al. 2014; Suomi et al. 2013). The prevalence of family violence in the gambler treatment seeking sample was high at 33.9% (11.0% victimisation only, 6.9% perpetration only, and 16.0% both victimisation and perpetration). The results also suggested that reciprocal victimisation and perpetration was commonly occurring in problem gambler households (Dowling et al. 2014). Consistent with some previous research (Kausch et al. 2006), the Australian female gamblers were 2.1 times more likely to report victimisation only and 1.6 times more likely to report both victimisation and perpetration than male gamblers (Dowling et al. 2014). The literature suggests some complexity when looking at gender in family violence and gambling. For example, Korman et al. (2008) found that females were more likely to report past year IPV minor injury perpetration (42% c.f. 21% of males), and severe injury perpetration (28% c.f. 7% of males) in a convenience sample of problem gamblers. Gender did not moderate the relationship between problem gambling severity, and minor or severe dating or marital violence perpetration in a large (n = 3,334) community sample in the USA (Afifi et al. 2010). Differences in measures of violence used and sample structures complicate comparisons and limit our understanding of the relationship between gender, family violence and gambling.

No research to date has explored, by gender, the phenomenon of family violence in the families of problem gamblers with, or without, the presence of dependent children. A high proportion of New Zealand children are considered vulnerable (Statistics New Zealand 2012). One-fifth of New Zealand households with children report each of the following factors related to poor child outcomes: having been a victim of crime in the past year; financial strain; living in a high deprivation area; and being isolated from one’s community (Statistics New Zealand 2012). By the time they are born, a third of New Zealand children are exposed to risk factors related to maternal wellbeing, financial stress and the home environment, that make them more likely to have poor health outcomes in childhood and later life (Morton et al. 2014). Indigenous New Zealand Māori and Pacific children experience greater exposure to vulnerability risk factors in the first nine months of life compared to New Zealand European and Asian children (Morton et al. 2014).

Further research is required if we are to address the particular issues that are associated with family violence in families with problem gamblers, when dependent children are also present. Thus, the present research aimed to examine: (1) the effect of the gender of the gambler on the relationship between having dependent children in the household and gambler violence perpetration or victimisation, and (2) how this interaction changed (both in size, direction and significance) as the contextual factors of family violence were taken into account.

## Methods

### Recruitment

As part of a larger study investigating family violence in people seeking assistance from gambling treatment services, a self-selected convenience sample of 164 treatment-seeking gamblers was recruited nationwide from three New Zealand national gambling treatment services from June 2013 to March 2015. The inclusion criteria were that participants were at least 18 years old and presented for counselling alone (i.e. without family members or any other person present). The latter criterion was to protect client safety from potential perpetrators when the invitation to participate in the family violence research was given.

### Procedure

Gamblers accessing the services who expressed an interest in taking part in the research were given or sent a participant information sheet by their counsellor, and signed a consent form for participation. Participants provided telephone contact details and indicated the most convenient time that they could be contacted by a researcher to complete a comprehensive questionnaire interview (taking 45–60 min), with the interviews conducted by telephone, in English, by trained researchers. Responses were recorded on paper with data subsequently entered into the statistical software package SPSS for analysis. Participants who completed the interview were sent a NZ$40 petrol voucher in recognition of their time.

### Measures

#### HITS Scale for family violence

The HITS Scale was developed to screen for women who were victims of family violence, comprising four items assessing physical and verbal abuse in a past 12-month timeframe (Sherin et al. 1998). The HITS has displayed high internal consistency (α = 0.80), good concurrent validity with other scales, and good construct validity. In regard to reliability, Cronbach’s alpha was 0.80 for the four item scale (Sherin et al. 1998). Each item is scored on a five-point frequency scale, from never (1) to frequently (5). Family violence is suspected when respondents score higher than 10 on the HITS. For the present study, a modified version of the HITS scale was used (see “Appendix”) whereby a question assessing sexual abuse was added from the Partner Violence Screen (Parker et al. 1993) and responses for each item were scored in a Yes/No format. Participants were asked to frame their response for the 12-month period before the start of their gambling counselling.

### Sociodemographic data

Age, gender, ethnicity, relationship status, employment status, whether receiving income support (including benefits, superannuation and student allowances), highest educational level attained and annual personal income data were collected.

### Psychosocial data

Measures of participant issues with alcohol, drugs, tobacco and mental health were collected via: the 10-item Alcohol Use Disorders Identification Test (AUDIT, Saunders et al. 1993); brief 10-item version of the Drug Abuse Screening Test (DAST, Skinner 1982); three single items on past and current tobacco use; and the Kessler-10 to measure general psychological distress (Kessler and Mroczek 1994). The presence of co-existing mental health issues, substance abuse and alcohol abuse (single items) in family members was also collected.

Various behavioural traits were measured via the anger and hostility subscales (each three-items) adapted from the Buss-Perry Aggression Questionnaire—short version (Bryant and Smith 2001; Buss and Perry 1992); emotion regulation using the impulse (difficulty controlling impulses), goals (difficulty engaging in goal-oriented behaviours) and strategies (lack of access to emotion regulation strategies) subscales of the Difficulties in Emotion Regulation Scale (DERS) (Gratz and Roemer 2004); and symptoms of distress using the short 30-item version of the Symptom Rating Test (SRT) (Kellner and Sheffield 1973). The SRT has four subscales assessing anxiety, depression, somatic symptoms and inadequacy symptoms.

Interpersonal support was measured using the short 12-item version of the Interpersonal Support Evaluation List (ISEL) (Cohen et al. 1985). There are three subscales, each comprising four items, measuring: appraisal support (support useful for self-evaluation such as esteem); belonging support (feeling a sense of social belonging); and tangible support (actual support from another person). Self-rated health and quality of life data were collected via single items.

### Gambling data

Gambling risk level was measured using the nine-item Problem Gambling Severity Index that assesses problem gambling in a past 12-month timeframe (PGSI, reliability Cronbach’s alpha score 0.93, Ferris and Wynne 2001).

Motivations to gamble were collected via the 15-item Gambling Motives Questionnaire adapted from the Drinking Motives Questionnaire (Cooper et al. 1992). It has three subscales: enhancement motives (internal positive reinforcement, i.e. gambling to increase positive emotions); coping motives (internal negative reinforcement, i.e. gambling to reduce or avoid negative emotions); and social motives (external positive reinforcement motives, i.e. gambling to increase social affiliation.

Gambling behaviour data via single item questions on gambling participation, length of gambling problem, legal consequences of gambling, self-exclusion from gambling venues and previous help-seeking behaviour such as counselling or medication were also collected.

## Data analysis

The effect of having dependent children (aged less than 18 years usually living at home) on ‘any family violence perpetration’ and ‘being a victim of any family violence’, and how this changes with the gender of the gambler, was investigated via four logistic multivariable models that took into account respectively, no adjusting factors (Model 1) and then, cumulatively, significant factors from socio-demographic (Model 2), psychosocial (Model 3) and gambling (Model 4) blocks of variables.

For each block, all covariates that had a p-value of ≤ 0.2 from simple models were considered (where numbers allowed) for selection into a multivariable block model that contained only variables from that block as well as the dependent children variable. Covariates investigated for each block are detailed in the preceding measures section.

To build each of the three multivariable block models for each violence type, a manual stepwise procedure was undertaken, with forward selection followed by possible backward selection. At each step, a likelihood ratio test was performed comparing the current model with a model that had the addition of one of the variables to be considered for selection. This was performed for each variable under consideration, and the variable that had the most significant p-value for the likelihood ratio test was then chosen for entry into the model for that step. At each step, backward selection was also performed by removing any variables one at a time that had p-values for the Wald Chi Square test ≥ 0.05. The procedure stopped when a variable had been removed and further forward selection failed to enter a new variable.

The adjustors ultimately selected for each individual multivariable block model are listed below:

- Sociodemographic factors: Asian and Māori ethnicity (violence perpetration block); Asian ethnicity and income support (victim of violence block).
- Psychosocial factors: Anger subscale of Aggression Questionnaire, strategies subscale of DERS, tangible support subscale of ISEL and family issue with drug use (violence perpetration block); Inadequacy subscale of SRT (victim of violence block).
- Gambling factors: Non-casino (i.e. pub and/or club) electronic gaming machines (EGMs) as main gambling activity, and receiving previous counselling or medication for gambling problems (violence perpetration block); Non-casino EGMs as main gambling activity (victim of violence block).

Finally, results from the three block models were combined in a new series of models by adding to Model 1 (‘dependent children’ covariate only): first variables from the sociodemographic block model (to give Model 2), then those from the psychosocial factors block model (to give Model 3), and finally those from the gambling block model (to give Model 4), with variables in each block being added together as a group. At each addition, any variables (apart from dependent children) that were found to have a Wald Chi Square test ≥ 0.05 were removed one at a time according to the size of their p-value.

P-values for each covariate in each final model are presented, together with odds ratios and 95% confidence intervals for each covariate category versus an appropriate reference group.

### Participants

Forty-three percent of the participants were female; about one-fifth each were of Māori or Asian ethnicity, about one-eighth were of Pacific descent, and about half were of European/Other descent. A majority were aged between 25 and 64 years, and about one-third were receiving income support. Thirty-seven percent of participants had a child/children aged less than 18 years living at home (dependent children). Full demographic details are presented in Table 1.

**Table 1:** Demographics

Demographic variable		Gamblers (n = 164)		With children^a^ (n = 60)		Without children^a^ (n = 104)	
		n	(%)	n	(%)	n	(%)
Gender							
Female		164	(42.7)	60	(51.7)	104	(37.5)
Male			(57.3)		(48.3)		(62.5)
Age group (years)							
20–24		162	(8.0)	59	(6.8)	103	(8.7)
25–44			(46.9)		(59.3)		(39.8)
45–64							
			(37.0)		(32.2)		(39.8)
65 +							
			(8.0)		(1.7)		(11.7)
Ethnicity							
Māori		162	(19.1)	59	(18.6)	103	(19.4)
Pacific			(12.3)		(27.1)		(3.9)
Asian			(17.9)		(16.9)		(18.4)
European/other			(50.6)		(37.3)		(58.3)
Relationship status							
Single, not in a relationship		164	(39.6)	60	(33.3)	104	(43.3)
In a relationship, but not living with partner			(6.7)		(8.3)		(5.8)
Married or defacto, living with partner			(51.2)		(56.7)		(48.1)
Married, not living with partner			(1.8)		(1.7)		(1.9)
Other			(0.6)		–		(1.0)
Employment status							
Employed		164	(66.5)	60	(75.0)	104	(61.5)
Unemployed			(18.9)		(18.3)		(19.2)
Student/homemaker/retired			(14.6)		(6.7)		(19.2)
Income support							
None		164	(59.8)	60	(50.0)	104	(65.4)
Benefit			(32.9)		(46.7)		(25.0)
Superannuation/student allowance			(7.3)		(3.3)		(9.6)
Highest qualification							
None/below secondary school level		163	(16.6)	59	(17.0)	104	(16.4)
Secondary school qualification			(39.3)		(44.1)		(36.5)
Trade/technical qualification			(11.0)		(6.8)		(13.5)
Undergraduate certificate/diploma			(18.4)		(20.3)		(17.3)
University degree or higher			(14.7)		(11.9)		(16.4)
Annual personal income							
≤ $20,000		164	(26.8)	60	(26.7)	104	(26.9)
$20,001–$40,000			(23.2)		(28.3)		(20.2)
$40,001–$60,000			(22.0)		(20.0)		(23.1)
$60,001–$80,000			(11.0)		(6.7)		(13.5)
$80,001–$100,000			(4.3)		(5.0)		(3.9)
> $100,000			(3.1)		(3.3)		(2.9)
Not reported			(9.8)		(10.0)		(9.6)

^a^With/without children aged less than 18 years usually living at home

## Results

### Family violence

Two-thirds (68%) of the female participants and almost half (45%) of the male participants with dependent children reported perpetrating some type of family violence in the prior 12 months. This compared with 44% of females and 31% of males without dependent children (Table 2). Similarly, three-quarters (77%) of females and 55% of males with dependent children reported being victims of family violence, compared with 41 and 39% respectively, without dependent children (Table 2). Chi square tests of independence between having dependent children and violence perpetration, and between having dependent children and being a victim of violence, revealed statistically significant differences only for females. Females with dependent children had greater odds for perpetrating violence (OR 2.72, 95% CI 1.02–7.27) and being victims of violence (OR 4.93, 95% CI 1.71–14.17) than females without dependent children.

**Table 2:** Family violence among gamblers by gender and presence of dependent children

	Types of violence^a^					Any violence (%)
	Physically hurt (%)	Insulted or talked down to (%)	Threatened with harm (%)	Screamed or cursed at (%)	Forced sexual activities (%)	
Violence perpetration in last 12 months						
Male						
No dependent children (n = 65)	6.2	23.1	4.6	27.7	–	30.8
Children (n = 29)	10.3	37.9	13.8	37.9	–	44.8
Total (n = 94)	7.4	27.7	7.5	30.9	–	35.1
Female						
No dependent children (n = 39)	7.7	33.3	12.8	38.5	–	43.6
Children (n = 31^b^)	6.4	41.9	3.3	64.5	–	67.7
Total (n = 70^b^)	7.1	37.1	8.7	50.0	–	54.3
Victim of violence in last 12 months						
Male						
No dependent children (n = 65)	7.7	27.7	4.6	33.9	3.1	38.5
Children (n = 29)	3.5	44.8	13.8	51.7	–	55.2
Total (n = 94)	6.4	33.0	7.5	39.4	2.1	43.6
Female						
No dependent children (n = 39)	5.1	30.8	12.8	35.9	2.6	41.0
Children (n = 31)	6.5	51.6	9.7	67.7	6.5	77.4
Total (n = 70)	5.7	40.0	11.4	50.0	4.3	57.1

^a^Multiple categories could be selected

^b^One missing data point for threatened with harm

The most prevalent type of violence (both for perpetrators and victims) was verbal abuse—‘insulted or talked down to’, and ‘screamed or cursed at’ with one-quarter to half of male and female participants reporting this. Chi Square tests of independence between gender and type of violence indicated a gender difference only for screaming or cursing (perpetration), with females more likely to do this (OR 2.24, 95% CI 1.18–4.26) than males (p = 0.01). The prevalence of perpetrating or threatening physical harm, or being a victim of these, was similar for males and females (6–11%). No participants reported perpetrating sexual violence; however, two percent of males and four percent of females reported being victims of sexual abuse. There was no statistical difference for a Fisher’s exact test of independence (p = 0.7). Results are shown in Table 2.

A majority of the family violence was between current or ex-partners with almost two-thirds of perpetrators (65%) and victims (62%) reporting this. About one-fifth of participants reported that the violence was to (16%) or from (20%) a parent. Overall, less than one-fifth of participants reported that the violence was to (18%) or from (12%) a son or daughter (age was not established and could include adult children). However, a greater proportion of females than males reported perpetrating, or being victims, of violence from a son or daughter. Other family members who were victims of (28%) or perpetrators of (21%) violence included wider family such as siblings, uncles/aunts and grandparents. Results are shown in Table 3.

**Table 3:** Relationship of gambler to those involved in violence, by gender

Relationship with victim or perpetrator^b^	Perpetrator %			Victim %		
	Male (n = 33)	Female (n = 38)	Total (n = 71^a^)	Male (n = 41)	Female (n = 40)	Total (n = 81^a^)
Current or ex-partner	66.7	63.2	64.8	65.9	57.5	61.7
Son or daughter	9.1	26.3	18.3	9.8	15.0	12.4
Parent	15.2	15.8	15.5	19.5	20.0	19.8
Any other family	27.3	15.8	21.1	31.7	25.0	28.4
Relation type not reported	3.0	2.6	2.8	4.9	2.5	3.7

^a^81 people reported being a victim of at least one type of family violence; 71 people reported perpetrating at least one type of violence

^b^Participants could select multiple relationships for a given type of violence; participants could also report more than one type of violence with the same or different relative(s)

### Associations with perpetrating family violence

Female participants with dependent children had more than four times the odds for perpetrating family violence (OR 4.72, 95% CI 1.89–11.84), compared with male participants without dependent children (see Model 1, Table 4). Significant sociodemographic adjustors for the association between ‘dependent children by gender of the gambler’ and perpetration of family violence, were found to be Asian ethnicity and Māori ethnicity; on adjusting for these, the association remained (OR 4.20, 95% CI 1.59–11.07) (see Model 2, Table 4). Carrying these sociodemographic factors forward to the next model where psychosocial factors were also adjusted for, significant psychosocial adjustors for the association were found to be anger, difficulties in emotion regulation strategies, tangible interpersonal support and having a family member with a drug issue in the past 12 months; note that Māori ethnicity became non-significant at this point and was removed (see Model 3, Table 4). The additional adjustment by psychosocial as well as sociodemographic factors resulted in the significance of the aforementioned odds ratio disappearing, as well as the significance of the overall effect of ‘dependent children by gender of gambler’ on violence perpetration.

**Table 4:** Series of regression models explaining/predicting ‘violence perpetration’

Covariates from individual block sub-models	Covariate category	Any violence perpetration % For covariate category	Model 1 (“Children” covariate only) n = 164		Model 2 (+ Socio-demographics) n = 162		Model 3 (+ Socio-demographics + Psycho-social factors) n = 155		Model 4 (+ Socio-demographics + Psycho-social factors + Gambling) n = 155	
			Odds ratio (95% CI)	p-value	Odds ratio (95% CI)	p-value	Odds ratio (95% CI)	p-value	Odds ratio (95% CI)	p-value
Children aged < 18 years usually living in household by Gender of Gambler	Male, no children (n = 65)	30.77	1 (reference)	0.01	1 (reference)	0.03	1 (reference)	0.26	1 (reference)	0.12
	Female, no children (n = 39)	43.59	1.74 (0.76–3.96)		1.58 (0.66–3.79)		1.24 (0.40–3.86)		1.35 (0.41–4.48)	
	Female, with children (n = 31)	67.74	4.72 (1.89–11.84)		4.20 (1.59–11.07)		3.20 (0.99–10.39)		4.36 (1.28–14.87)	
	Male, with children (n = 29)	44.83	1.83 (0.74–4.50)		2.47 (0.93–6.52)		1.76 (0.51–6.09)		1.77 (0.49–6.46)	
Socio-demographics (block 1)										
Asian	No (n = 133)	49.62			1 (reference)	0.02	1 (reference)	0.001	1 (reference)	0.001
	Yes (n = 29)	17.24	–		0.28 (0.096–0.81)		0.12 (0.03–0.42)		0.12 (0.03–0.44)	
Maori	No (n = 131)	37.40			1 (reference)	0.01				
	Yes (n = 31)	70.97	–		3.30 (1.33–8.16)		(Not significant here)		(Not significant here)	
Psycho-social factors (block 2)										
Aggression questionnaire— Anger subscale (> 3.5, <=6 vs <=3.5)	≤ 3.5 (n = 41)	17.07					1 (reference)	0.001	1 (reference)	0.002
	>3.5, ≤ 6 (n = 44)	40.91	–		–		2.84 (0.81–9.96)		2.40 (0.64–8.92)	
	> 6, ≤ 10 (n = 47)	51.06	–		–		8.85 (2.29–34.26)		7.54 (1.86–30.59)	
	> 10 (n = 32)	68.75	–		–		20.50 (4.32–97.24)		20.69 (4.11–104.01)	
Difficulties in emotion regulation—Strategies	≤ 10 (n = 46)	28.26					1 (reference)	0.002	1 (reference)	0.003
	> 10, ≤ 15 (n = 38)	60.53	–		–		3.76 (1.11–12.76)		3.30 (0.93–11.70)	
	> 15, ≤ 22 (n = 44)	29.55	–		–		0.30 (0.09–1.05)		0.25 (0.07–0.93)	
	> 22 (n = 36)	61.11	–		–		1.43 (0.37–5.55)		1.28 (0.32–5.22)	
Drugs issue in family/whanau in last 12 months	No (n = 137)	37.96					1 (reference)	0.02	1 (reference)	0.03
	Yes (n = 23)	69.57	–		–		4.53 (1.27–16.24)		4.58 (1.20–17.44)	
Interpersonal Support Evaluation List—Tangible subscale	≥ 10, < 12 (n = 32)	53.13					1 (reference)	0.01	1 (reference)	0.02
	< 7 (n = 33)	54.55	–		–		0.53 (0.13–2.14)		0.66 (0.16–2.81)	
	≥ 7, < 10 (n = 33)	30.30	–		–		0.09 (0.02–0.40)		0.10 (0.02–0.44)	
	≥ 12 (n = 63)	38.10	–		–		0.33 (0.11–1.01)		0.35 (0.11–1.11)	
Gambling (block 3)										
Received counselling or medication for gambling in last 12 months	No (n = 127)	36.22							1 (reference)	0.03
	Yes but not now (n = 21)	66.67	–		–		–		3.81 (1.07–13.59)	
	Yes currently (n = 16)	68.75	–		–		–		4.61 (0.92–23.10)	
Adjusted R-squared			0.09		0.22		0.49		0.53	

The gambling “block” model for violence perpetration (which just had covariates “Children aged < 18 years usually living in household by Gender of Gambler”, “Received Counselling or medication for gambling in last 12 months”, and “Pub or club EGMs main type”) had an odds ratio for violence perpetration comparing “Pub or club EGMs is the main type” to “Pub or club EGMs is NOT the main type” of 2.25 (1.09–4.64) with a significant p-value of 0.03. Had we included “Pub or club EGMs main type” in Model 4 for violence perpetration, which takes into account socio-demographic and psychosocial factors, we would have reduced the “Pub or club EGMs main type” odds ratio to an insignificant (with p = 0.9) 1.07 (0.39–2.93); it was therefore not included in Model 4

Carrying the significant sociodemographic and psychosocial factors forward to the fourth model where adjustment for gambling factors was added, the significant gambling-related adjustor for the association was having received counselling or medication for gambling in the last 12 months. Non-casino (i.e. pub and/or club) electronic gaming machines (EGMs) as main gambling activity, originally part of the multivariable gambling block model, became non-significant at this point, and was removed from this model (see Model 4, Table 4). Adjustment by sociodemographic, psychosocial and gambling factors combined then resulted in the odds ratio for perpetration of violence comparing female participants with children, to male participants without children, becoming significant again (OR 4.36, 95% CI 1.28–14.87); however, the overall effect of ‘dependent children by gender of gambler’ on violence perpetration remained non-significant (see Model 4, Table 4).

On further investigation, an identical model to Model 4 in Table 4, except with ‘dependent children’ in place of ‘dependent children by gender of gambler’ as the primary explanatory variable was run. This resulted in gamblers with dependent children being estimated to have 2.56 times (95% CI 1.04–6.30) the odds of perpetrating family violence, than gamblers without dependent children (p-value 0.04). Similarly for Model 3 in Table 4, replacing ‘dependent children by gender of gambler’ with ‘dependent children’, gave an estimate of gamblers with dependent children having 2.24 times (95% CI 0.92–5.43) the odds of perpetrating violenInsert Table 4 about here (landscape table so has been submitted separately from manuscript). ce than those without dependent children, though this failed to reach significance (p-value 0.07).

### Associations with being a victim of family violence

Female participants with dependent children had more than five times the odds of being a victim of family violence (OR 5.49, 95% CI 2.06–14.60), compared to male participants without dependent children (see Model 1, Table 5). Significant sociodemographic adjustors for the association between ‘dependent children by gender of the gambler’ and being a victim of family violence, were found to be Asian ethnicity and income support; on adjusting for these, the association remained, and the odds ratio increased to 6.77 (95% CI 2.39–19.22) (see Model 2, Table 5). Carrying these sociodemographic factors forward to the next model where psychosocial factors were also adjusted for, a significant psychosocial adjustor for the association was found to be symptoms of inadequacy (see Model 3, Table 5). The additional adjustment by psychosocial as well as sociodemographic factors did not affect the significance of the odds ratio, though it was slightly reduced compared to Model 2 (OR 5.97, 95% CI 2.06–17.34) (see Model 3, Table 5).

**Table 5:** Series of logistic regression models explaining/predicting ‘violence victimisation’

Covariates from individual block sub-models	Covariate category	Any violence victimisation % For covariate category	Model 1 (“Children” covariate only) n = 164		Model 2 (+ Socio-demographics) n = 162		Model 3 (+ Socio-demographics + Psycho-social factors) n = 159	
			Odds ratio (95% CI)	p-value	Odds ratio (95% CI)	p-value	Odds ratio (95% CI)	p-value
Children aged < 18 years usually living in household by Gender of Gambler	Male, no children (n = 65)	38.46	1 (reference)	0.005	1 (reference)	0.002	1 (reference)	0.005
	Female, no children (n = 39)	41.03	1.13 (0.50–2.50)		1.01 (0.43–2.36)		0.95 (0.39–2.33)	
	Female, with children (n = 31)	77.42	5.49 (2.06–14.60)		6.77 (2.39–19.22)		5.97 (2.06–17.34)	
	Male, with children (n = 29)	55.17	1.97 (0.81–4.78)		2.41 (0.92–6.34)		2.16 (0.78–6.01)	
Socio-demographics (block 1)								
Asian	No (n = 133)	54.89	–		1 (reference)	0.005	1 (reference)	0.02
	Yes (n = 29)	27.59	–		0.25 (0.09–0.66)		0.30 (0.10–0.85)	
Income support	No benefit (n = 99)	53.54	–		1 (reference)	0.009	1 (reference)	0.01
	Support/NZ Super/Student (n = 65)	43.08	–		0.37 (0.18–0.78)		0.38 (0.17–0.81)	
Psycho-social factors (block 2)								
Symptom rating scale—Inadequacy	≤ 3 (n = 61)	36.07	–		–		1 (reference)	0.02
	> 3, ≤ 5 (n = 22)	31.82	–		–		0.86 (0.28–2.69)	
	> 5, ≤ 8 (n = 39)	64.10	–		–		3.00 (1.17–7.66)	
	> 8 (n = 39)	64.10	–		–		2.99 (1.20–7.44)	
Adjusted R-squared			0.12		0.22		0.28	

Note, for the model predicting violence victimisation, no gambling block variables were able to be added

The gambling “block” model for violence victimisation (which just had covariates “Children aged < 18 years usually living in household by Gender of Gambler” and “Pub or club EGMs main type”) had an odds ratio for violence victimisation comparing “Pub or club EGMs is the main type” to “Pub or club EGMs is NOT the main type” of 2.37 (1.21–4.65) with a significant p-value of 0.01. Had we added “Pub or club EGMs main type” to Model 3 for violence victimisation which takes into account socio-demographic and psychosocial factors we would have reduced the odds ratio above to an insignificant (with p = 0.30) 1.51 (0.70–3.29)

Carrying the significant sociodemographic and psychosocial factors forward to a fourth model where adjustment for gambling factors was added, a significant gambling adjustor for the association was found to be non-casino EGMs as main gambling activity; however, as with the model for perpetration, this adjustor became non-significant at this point and was removed from this model. As no gambling factors could, therefore, be added, this model was discarded. Thus, the final model for violence victimisation, was Model 3 as shown in Table 5.

## Discussion

The aim of the current research was to examine the effect of the gender of the gambler on the relationship between having dependent children in the household and gambler violence perpetration or victimisation, and then how this interaction changed as the contextual factors of family violence were taken into account.

The presence of dependent children was associated with a greater proportion of female gamblers reporting both perpetration of, and being a victim of, family violence compared with male gamblers. The major type of violence was verbal—‘insulted or talked down to’ and ‘screamed or cursed at’, with females significantly more likely to scream or curse at someone (generally a current or ex-partner, followed by a parent) than males. These findings are of particular interest as this is not an area that has previously attracted research attention. Verbal aggression is an understudied phenomenon in IPV research. Straus and Sweet (1992), using a nationally representative data set in the USA, found equal frequency of verbal aggression in males and females, and male–female (couple) reports were highly correlated. Stets and Straus (1990), using the same national dataset, argued that verbal and physical aggression are separate dimensions of aggression; each having their own predictor variables. Elevated verbal aggression among females in gambling populations, as suggested here, underscores the importance of continuing to disaggregate and examine family violence typology and trajectories with families affected by gambling.

Being a female gambler living with dependent children was associated with over five times the odds of also being a victim of family violence amongst help-seeking gamblers, compared to male gamblers without dependent children. Importantly, this finding remained even after sociodemographic and psychosocial factors known to be associated with violence were adjusted for, and once gambling factors were considered. Our final adjusted estimate of the odds was that females with dependent children were six times more likely than males without dependent children to be victims of family violence (Model 4, Table 5). The persistence of this effect, despite adjustment for known contextual factors, indicates a strong gender dimension to the effect of having dependent children on being a victim of family violence among gamblers. Why is it that females living with dependent children are especially vulnerable? This question warrants further research in regard to the particular gendered social and psychological processes at work, and highlights the necessity for interventions to reduce victimisation that involve female gamblers with dependent children. In this study, over two-thirds of female gambler participants with dependent children lived in homes where violence was occurring; 68% where violence was perpetrated by the female gambler, and 77% where the female gambler was a victim of violence. Thus, children in such homes were subject to an environment with violence occurring.

In addition, gambling predominantly on non-casino EGMs became non-significant when added to a model that had already taken into account psychosocial and sociodemographic factors. This indicates that it is not preference for gambling on non-casino EGMs itself that is related to increased violence victimisation, but rather the possible association of non-casino EGMs with sociodemographic (Asian ethnicity, income support) and/or psychosocial factors (symptoms of inadequacy). Interventions to reduce violence victimisation among those whose main form of gambling is non-casino EGMs could be enhanced by further investigation of the impact of these factors on victimisation.

Being a female gambler and living with dependent children was significantly associated with more than four times the odds for perpetrating family violence amongst help-seeking gamblers, compared with being male and not living with dependent children, even after adjusting for significant sociodemographic factors. However, once psychosocial factors were also taken into account, this effect disappeared. This suggests that it is not the combination of female gender and having dependent children per se that is related to increased violence perpetration but the association of that combination with other contextual factors that leads to the higher risk for perpetrating family violence. In their Australian study, Dowling et al. (2014) discuss their findings of elevated odds of victimisation, and combined victimisation and perpetration among female gamblers commenting that “this group of service users should be specifically targeted for screening, assessment and management” (p. 1716). The current results suggest that interventions targeted to female gamblers with dependent children that focus on addressing broader family issues such as drug use and the availability of tangible support, in addition to emotion regulation strategies could potentially reduce the risk of violence perpetration by female gamblers with dependent children.

Māori ethnicity also became insignificant in relation to violence perpetration once psychosocial factors were adjusted for. Previous research has identified that Māori have a higher risk of intimate partner violence than non-Māori (Family Violence Death Review Committee 2014; Fanslow et al. 2010; Koziol-McLain et al. 2010; Marie et al. 2008; Morris et al. 2003). Similarly, Morrison and Wilson (2015), in their qualitative study of Indigenous women’s gambling, reported that Māori women use gambling to escape from the tedium of their family lives including the care of children and grandchildren, but when the gambling becomes problematic this can lead to violence within the home. The current model for violence perpetration shows that it is not Māori ethnicity per se that is related to an increase in violence perpetration, but rather the association of ethnicity with psychosocial factors (e.g. tangible social support) that leads to the higher risk for perpetrating family violence.

As with victimisation, preference for gambling on non-casino EGMs became non-significant when added to a model that had already taken into account psychosocial and sociodemographic factors. This indicates that interventions to reduce violence perpetration among those whose main form of gambling is non-casino EGMs could also be enhanced by further investigation of the impact of these factors on perpetration.

Additional adjustment for having received counselling or medication for gambling in the last 12 months resulted in the association of females with dependent children and violence perpetration re-appearing as significant. Being currently in counselling for gambling or receiving counselling in the past was associated with increased risk of perpetrating violence. It seems likely, therefore, that adjusting for counselling amounted to an adjustment for the severity of violence, meaning that those with more severe problems with violence perpetration were more likely to be receiving counselling or medication. The overall effect of the variable of interest was non-significant, showing that even though, in isolation, the particular comparison investigated was significant, as a whole, comparisons between the different categories of ‘dependent children by gender of gambler’ were non-significant. Thus, as the overall effect of the variable of interest was non-significant, and the adjustment for counselling seems to be somewhat circular, the authors do not believe the specific effect of receiving counselling by females with dependent children to be useful.

From this study, some unexpected or complex relationships were observed between the categories of the adjustors and violence perpetration. For example, regarding tangible support, it was only the second quantile compared to the third quantile that showed an association with a lower level of violence perpetration, whilst for strategies in emotion regulation, it was only the second quantile compared to the first quantile that was associated with a higher level of violence perpetration. Thus, further research is required to fully understand these relationships and their implications.

Another finding was that Asian ethnicity was associated with a reduction in violence perpetration. This was unexpected as the limited research on problematic gambling and family violence amongst Asian people indicates the existence of a positive (i.e. increased violence) relationship (Bhuyan et al. 2005; Keen et al. 2015; Tse et al. 2004). A possible explanation for our result could be cultural norms preventing open discussion of issues that could be considered shameful to the family (the need to ‘save face’ or to minimise/hide problems) when answering questions on perpetrating family violence, particularly if the family included children; this may have led to conservative disclosure of violent behaviour in our study (Au 1998; Ho 1990; Tse et al. 2004). It should be kept in mind that the results for all the adjustment factors are not estimated independent effects that have themselves been adjusted for variables relevant to them—they are only present as adjustors in the relationship of the primary variable (dependent children by gender of gambler) with violence perpetration.

### Limitations

Participants were a self-selected convenience sample of gamblers seeking professional help in New Zealand. The study sample was demographically fairly similar to the profile of gamblers accessing face-to-face gambling treatment services at the time of recruitment (Ministry of Health 2016); however, the study included a greater proportion of Asian participants and slightly more females. Thus, the study findings are reasonably, though not completely, representative of the gambling treatment-seeking population at the time.

Family violence in this study was broadly defined and included perpetration of, or being a victim of, physical, verbal, emotional or sexual abuse, together considered to be ‘family violence’. It was assessed using a modified version of the HITS Scale, which is a weakness of the study since violence was recorded as present or absent without frequency of occurrence, meaning it could have taken place only once or frequently in the prior 12 months. This may mean there is some over-estimation of violence. Additionally, a measure of financial abuse was not included in the modified version of the HITS Scale; as financial difficulties invariably are associated with problematic gambling, this could have been an important area for examination. A further weakness is that effects of violent behaviour such as fear and intimidation, were not captured. Behavioural effects of violence give a perpetrator power and control over the victim, which is a major part of sustained coordinated violence (Adams 2008).

The current study findings may be specific to the population of treatment-seeking gamblers and may not be representative of people in the general population who are directly, or indirectly, experiencing negative effects from gambling, and who have not accessed treatment services for gambling. As previously mentioned, the severity and frequency of the violence was not measured in the current study, which may also have affected the results, compared to other studies.

## Conclusion

This study has shown that the relationship between having dependent children and being a victim of family violence was gender-related, with female gamblers living with dependent children reporting more family violence perpetration and victimisation than male gamblers living with dependent children. Additionally, female gamblers with dependent children had greater odds of being a victim of family violence than male gamblers without dependent children. The implications of this finding are that children living in homes where the mother is a gambler, have a higher risk of living in a household within which family violence is occurring, increasing their vulnerability to poorer health outcomes. Thus, as a harm minimisation measure, it would be prudent for a coordinated approach to be developed between different health and social service sectors for potential early identification of family violence. In particular, it would be useful for gambling treatment services to routinely screen for family violence, particularly in households with dependent children, so that clients can be facilitated, as necessary, to relevant family violence services for assistance. Conversely, family violence services may benefit from screening for problematic gambling. In an ideal world, there would be a collaborative inter-agency and case management approach in order to identify family violence amongst people, particularly those with dependent children, who are affected by gambling problems; this could improve the prospects and outcomes for those people.

## Appendix: Modified HITS scale and sexual abuse question

### Victimisation

In the last 12 months before you started counselling on [date], has a current or ex-partner or a family member:

- Physically hurt you?
- Insulted or talked down to you?
- Threatened you with harm?
- Screamed or cursed at you?
- Forced you to have sexual activities?

### Perpetration

In the last 12 months before you started counselling on [date], have you…

- Physically hurt a current or ex-partner or family member?
- Insulted or talked down to a current or ex-partner or family member?
- Threatened a current or ex-partner or family member with harm?
- Screamed or cursed at a current or ex-partner or family member?
- Forced a current or ex-partner or family member to have sexual activities?

#### Publisher’s Note

Springer Nature remains neutral with regard to jurisdictional claims in published maps and institutional affiliations.

## References

[CR1] Abbott, M., Bellringer, M., Garrett, N., & Mundy-McPherson, S. (2014). *New Zealand 2012 National Gambling Study: Gambling harm and problem gambling. Report number 2*. Auckland: Auckland University of Technology, Gambling and Addictions Research Centre.

[CR2] Abbott, M., & Volberg, R. (1991). *Gambling and problem gambling in New Zealand. Research Series No. 12*. Wellington: Department of Internal Affairs.

[CR3] Abbott M, Volberg R (1996). The New Zealand national survey of problem and pathological gambling. Journal of Gambling Studies.

[CR4] Abbott, M., & Volberg, R. (2000). *Taking the pulse on gambling and problem gambling in New Zealand: A report on phase one of the 1999 National Prevalence Survey. Report number three of the New Zealand Gaming Survey*. Wellington: Department of Internal Affairs.

[CR5] Adams PJ (2008). Fragmented intimacy: Addiction in a social world.

[CR6] Afifi TO, Brownridge DA, MacMillan H, Sareen J (2010). The relationship of gambling to intimate partner violence and child maltreatment in a nationally representative sample. Journal of Psychiatric Research.

[CR7] Au, P. (1998). *A study of domestic violence within the Chinese community in Auckland*. The University of Auckland: Dissertation submitted in partial fulfilment of the requirements for the degree of Master of Education (Counselling).

[CR8] Bhuyan R, Mell M, Senturia K, Sullivan M, Shiu-Thornton S (2005). “Women must endure according to their karma” Cambodian immigrant women talk about domestic violence. Journal of Interpersonal Violence.

[CR03] Bland RC, Newman SC, Orn H, Stebelsky G (1993). Epidemiology of pathological gambling in Edmonton. Canadian Journal of Psychiatry.

[CR9] Bohn DK, Tebben JG, Campbell JC (2004). Influences of income, education, age, and ethnicity on physical abuse before and during pregnancy. Journal of Obstetric, Gynecologic, and Neonatal Nursing.

[CR10] Bryant FB, Smith BD (2001). Refining the architecture of aggression: A measurement model for the Buss-Perry Aggression Questionnaire. Journal of Research in Personality.

[CR11] Buss AH, Perry M (1992). The aggression questionnaire. Journal of Personality and Social Psychology.

[CR12] Clarke D, Abbott M, Tse S, Townsend S, Kingi P, Manaia W (2006). Associations with pathological gambling in a New Zealand urban sample. New Zealand Journal of Psychology.

[CR13] Cohen S, Mermelstein R, Kamarck T, Hoberman HM, Sarason IG, Sarason BR (1985). Measuring the functional components of social support. Social support: Theory, research and applications.

[CR14] Cooper ML, Russell M, Skinner JB, Windle M (1992). Development and validation of a three-dimensional measure of drinking motives. Psychological Assessment.

[CR15] Delfabbro P (2000). Gender differences in Australian gambling: A critical summary of sociological and psychological research. Australian Journal of Social Issues.

[CR16] Dowling N, Jackson AC, Suomi A, Lavis T, Thomas SA, Patford J (2014). Problem gambling and family violence: Prevalence and patterns in treatment-seekers. Addictive Behaviors.

[CR17] Dowling N, Smith D, Thomas T (2009). The family functioning of female pathological gamblers. International Journal of Mental Health and Addiction.

[CR18] Dowling N, Suomi A, Jackson A, Lavis T, Patford J, Cockman S (2016). Problem gambling and intimate partner violence: A systematic review and meta-analysis. Trauma, Violence, & Abuse.

[CR19] Dyall L (2007). Gambling, social disorganisation and deprivation. International Journal of Mental Health and Addiction.

[CR20] Family Violence Death Review Committee. (2014). *Fourth annual report: January 2013 to December 2013*. Wellington: Family Violence Death Review Committee.

[CR21] Fanslow J, Robinson E, Crengle S, Perese L (2010). Juxtaposing beliefs and reality: Prevalence rates of intimate partner violence and attitudes to violence and gender roles reported by New Zealand women. Violence Against Women.

[CR22] Ferris, J. A., & Wynne, H. J. (2001). *The Canadian problem gambling index: Final report*. Ottawa, ON: Canadian Centre on Substance Abuse.

[CR23] Fox GL, Benson ML (2006). Household and neighborhood contexts of intimate partner violence. Public Health Reports.

[CR24] Gratz KL, Roemer L (2004). Multidimensional assessment of emotion regulation and dysregulation: Development, factor structure, and initial validation of the difficulties in Emotion Regulation Scale. Journal of Psychopathology and Behavioral Assessment.

[CR25] Griffiths, M., & Delfabbro, P. (2001). The biopsychosocial approach to gambling: Contextual factors in research and clinical interventions. *Journal of Gambling Issues,* (5). doi:10.4309/jgi.2001.5.1

[CR26] Gulliver, P., & Fanslow, J. L. (2013). *Family violence indicators: Can administrative data sets be used to measure trends in family violence in New Zealand*? Wellington: SuPERU: Families Commission.

[CR27] Heise LL, Kotsadam A (2015). Cross-national and multilevel correlates of partner violence: An analysis of data from population-based surveys. The Lancet Global Health.

[CR28] Ho CK (1990). An analysis of domestic violence in Asian American communities: A multicultural approach to counseling. Women & Therapy.

[CR29] Kalischuk RG, Nowatzki N, Cardwell K, Klein K, Solowoniuk J (2006). Problem gambling and its impact on families: A literature review. International Gambling Studies.

[CR30] Kausch O, Rugle L, Rowland DY (2006). Lifetime histories of trauma among pathological gamblers. The American Journal on Addictions.

[CR31] Keen B, Pickering D, Wieczorek M, Blaszczynski A (2015). Problem gambling and family violence in the Asian context: A review. Asian Journal of Gambling Issues and Public Health.

[CR32] Kellner R, Sheffield BF (1973). A self-rating scale of distress. Psychological Medicine.

[CR33] Kessler, R., & Mroczek, D. (1994). *Final versions of our non*-*specific Psychological Distress Scale*. Surrey Research Center of the Institute for Social Research, University of Michigan.

[CR34] Korman LM, Collins J, Dutton D, Dhayananthan B, Littman-Sharp N, Skinner W (2008). Problem gambling and intimate partner violence. Journal of Gambling Studies.

[CR35] Kourgiantakis T, Saint-Jacques M-C, Tremblay J (2013). Problem gambling and families: A systematic review. Journal of Social Work Practice in the Addictions.

[CR36] Koziol-McLain J, Garrett N, Fanslow J, Hassall I, Dobbs T, Lovell V (2010). A randomized controlled trial of a brief emergency department intimate partner violence screening intervention. Annals of Emergency Medicine.

[CR37] Kruger T, Pitman M, Grennell D, McDonald T, Mariu D, Pomare A (2004). Transforming whānau violence: A conceptual framework. An updated version of the report from the former Second Māori Taskforce on Whānau Violence.

[CR02] Lesieur HR, Rothschild J (1989). Children of gamblers anonymous members. Journal of Gambling Behavior.

[CR38] Lievore D, Mayhew P, Mossman E (2007). The scale and nature of family violence in New Zealand: A review and evaluation of knowledge.

[CR01] Lorenz VC, Shuttlesworth DE (1983). The impact of pathological gambling on the spouse of the gambler. Journal of Community Psychology.

[CR39] Lown EA, Schmidt LA, Wiley J (2006). Interpersonal violence among women seeking welfare: Unraveling lives. American Journal of Public Health.

[CR40] Marie D, Fergusson DM, Boden JM (2008). Ethnic identity and intimate partner violence in a New Zealand birth cohort. Social Policy Journal of New Zealand.

[CR41] McMillen J, Marshall D (2004). Victorian longitudinal community attitudes survey on gambling.

[CR42] Ministry of Health (2006). Problem gambling in New Zealand: Analysis of the 2002/03 New Zealand health survey.

[CR43] Ministry of Health (2008). A portrait of health: Key results of the 2006/07 New Zealand health survey.

[CR44] Ministry of Health. (2016). *Intervention client data*. http://www.health.govt.nz/our-work/mental-health-and-addictions/problem-gambling/service-user-data/intervention-client-data#ppgm. Accessed 29 Sept 2017.

[CR45] Morris, A., Reilly, J., Berry, S., & Ransom, R. (2003). *New Zealand national survey of crime victims 2001*. Wellington: Ministry of Justice. https://library.nzfvc.org.nz/cgi-bin/koha/opac-detail.pl?biblionumber=2942. Accessed 29 Sept 2017.

[CR46] Morrison L, Wilson D (2015). A family affair: Indigenous women’s gambling journey. International Journal of Mental Health and Addiction.

[CR47] Morton, S. M. B., Atatoa Carr, P. E., Grant, C. C., Berry, S. D., Marks, E. J., Chen, X. M.-H., et al. (2014). *Growing Up in New Zealand: A longitudinal study of New Zealand children and their families. Vulnerability Report 1: Exploring the definition of vulnerability for children in their first 1000* *days*. Auckland: The University of Auckland.

[CR48] Parker B, McFarlane J, Soeken K, Torres S, Campbell D (1993). Physical and emotional abuse in pregnancy: A comparison of adult and teenage women. Nursing Research.

[CR49] Perese, L. (2009). *You bet your life… and mine! Contemporary Samoan gambling in New Zealand*. University of Auckland: Thesis submitted in partial fulfilment of the requirements for the degree of Doctor of Philosophy.

[CR50] Raylu N, Oei TP (2004). Role of culture in gambling and problem gambling. Clinical Psychology Review.

[CR51] Rintoul AC, Livingstone C, Mellor AP, Jolley D (2013). Modelling vulnerability to gambling related harm: How disadvantage predicts gambling losses. Addiction Research & Theory.

[CR52] Saunders JB, Aasland OG, Babor TF, De la Fuente JR, Grant M (1993). Development of the alcohol use disorders identification test (AUDIT): WHO collaborative project on early detection of persons with harmful alcohol consumption-II. Addiction.

[CR53] Sherin KM, Sinacore JM, Li X-Q, Zitter RE, Shakil A (1998). HITS: a short domestic violence screening tool for use in a family practice setting. Family Medicine.

[CR54] Skinner HA (1982). The drug abuse screening test. Addictive Behaviors.

[CR55] Statistics New Zealand (2012). Vulnerable children and families: Some findings from the New Zealand general social survey.

[CR56] Stets JE, Straus MA, Straus MA, Gelles RJ (1990). Gender differences in reporting marital violence and its medical and psychological consequences. Physical violence in American families: Risk factors and adaptations in American families.

[CR57] Straus MA, Sweet S (1992). Verbal/symbolic aggression in couples: Incidence rates and relationships to personal characteristics. Journal of Marriage and Family.

[CR58] Suomi A, Jackson AC, Dowling NA, Lavis T, Patford J, Thomas SA, Koziol-McLain J (2013). Problem gambling and family violence: Family member reports of prevalence, family impacts and family coping. Asian Journal of Gambling Issues and Public Health.

[CR59] Tse, S., Wong, J., & Kim, H. (2004). A public health approach for Asian people with problem gambling in foreign countries. *Journal of Gambling Issues* (12). doi:10.4309/jgi.2004.12.13

[CR60] United Nations. (2014). *Review and appraisal of the implementation of the Beijing Declaration and Platform for Action and the outcomes of the twenty*-*third special session of the General Assembly*. United Nations: Economic and Social Council. https://reliefweb.int/sites/reliefweb.int/files/resources/N1469675.pdf. Accessed 29 Sept 2017.

[CR61] Volberg RA, Abbott M (1997). Gambling and problem gambling among indigenous peoples. Substance Use and Misuse.

[CR62] Watene, N., Thompson, K., Barnett, A., Balzer, M., & Turinui, M. (2007). *Whakatau Mai Ra: The impacts of gambling for Māori communities*—*A national Māori collaborative approach*. Waikato: Te Runanga o Kirikiriroa Trust Inc, Pou Tuia Rangahau (Research and Development).

[CR63] Wenzel SL, Tucker JS, Elliott MN, Marshall GN, Williamson SL (2004). Physical violence against impoverished women: A longitudinal analysis of risk and protective factors. Women’s Health Issues.

